# From positron emission tomography to cell analysis of the 18-kDa Translocator Protein in mild traumatic brain injury

**DOI:** 10.1038/s41598-021-03416-3

**Published:** 2021-12-14

**Authors:** Clément Delage, Nicolas Vignal, Coralie Guerin, Toufik Taib, Clément Barboteau, Célia Mamma, Kahina Khacef, Isabelle Margaill, Laure Sarda-Mantel, Nathalie Rizzo-Padoin, Fortune Hontonnou, Catherine Marchand-Leroux, Dominique Lerouet, Benoit Hosten, Valérie Besson

**Affiliations:** 1 Faculté de Pharmacie de Paris, Université Paris Descartes, EA4475 - Pharmacologie de la circulation cérébrale, Paris, France; 2 Faculté de Pharmacie de Paris, Université de Paris, Inserm UMR-S 1144 – Optimisation Thérapeutique en Neuropsychopharmacologie, 4 avenue de l’Observatoire, 75006 Paris, France; 3 Assistance Publique – Hôpitaux de Paris (AP-HP), Service de Médecine Nucléaire, Hôpital Lariboisière, Paris, France; 4 Université de Paris, Institut de Recherche Saint-Louis, Unité Claude Kellershohn, Paris, France; 5 Université de Paris, Innovative Therapies in Haemostasis, Inserm, 75006 Paris, France; 6 Institut Curie, Cytometry Core, 75005 Paris, France; 7 Université de Paris, Inserm UMS 3612 CNRS – US25 Inserm –Faculté de Pharmacie de Paris, Paris, France; 8 Faculté de Pharmacie de Paris, Université de Paris, Inserm UMR-S 1140, Paris, France; 9 CHU de Martinique, Service Pharmacie, Hôpital Pierre Zobda-Quitman, Fort-de-France, France; 10 Université de Paris, Inserm UMR-S 942, Hôpital Lariboisière, Paris, France; 11 Assistance Publique – Hôpitaux de Paris (AP-HP), Service Pharmacie, Hôpital Saint-Louis, Paris, France

**Keywords:** Neurology, Neuroscience, Cellular neuroscience, Glial biology, Molecular neuroscience, Neuroimmunology

## Abstract

Traumatic brain injury (TBI) leads to a deleterious neuroinflammation, originating from microglial activation. Monitoring microglial activation is an indispensable step to develop therapeutic strategies for TBI. In this study, we evaluated the use of the 18-kDa translocator protein (TSPO) in positron emission tomography (PET) and cellular analysis to monitor microglial activation in a mild TBI mouse model. TBI was induced on male Swiss mice. PET imaging analysis with [^18^F]FEPPA, a TSPO radiotracer, was performed at 1, 3 and 7 days post-TBI and flow cytometry analysis on brain at 1 and 3 days post-TBI. PET analysis showed no difference in TSPO expression between non-operated, sham-operated and TBI mice. Flow cytometry analysis demonstrated an increase in TSPO expression in ipsilateral brain 3 days post-TBI, especially in microglia, macrophages, lymphocytes and neutrophils. Moreover, microglia represent only 58.3% of TSPO^+^ cells in the brain. Our results raise the question of the use of TSPO radiotracer to monitor microglial activation after TBI. More broadly, flow cytometry results point the lack of specificity of TSPO for microglia and imply that microglia contribute to the overall increase in TSPO in the brain after TBI, but is not its only contributor.

## Introduction

Traumatic brain injury (TBI) is a growing public health concern for high-income countries, as a leading cause of mortality and disability in the under 40^1^. TBI is estimated to cause 282 000 hospitalizations and 56 000 deaths in the USA each year^2^, and to cost between 60.4 and 221 billion dollars for one year only in the USA^1^. Moreover, TBI is responsible for important sequelae in surviving patients. Indeed, studies linked TBI to neurodegenerative diseases^3^. These last years, neuroinflammation (NI) consecutive to the TBI has increasingly been recognized as the source of these long-term neurological disorders^4^.

NI is both a short and long term consequence of TBI, whether it is mild, moderate or severe, in humans or in animal models^5,6^. NI has been shown to last for decades after the injury^7^ and many studies linked its chronicity to white matter injuries and neurodegenerative diseases^4,8^. Microglia, as the main immune cells in the central nervous system (CNS), appear to be key cellular mediators of NI, both as promoting and repressing it. Microglia has been classically described through its two activation phenotypes: the pro-inflammatory (also called M1) and the anti-inflammatory (also called M2)^9^. However, it is currently recognized that microglia display a wide range of reaction states that are far more complex than this M1/M2 classification^10^. Microglia might form a community of cells in which each member or subtype displays distinct properties, performs unique physiological functions, depending on their regional distribution, gene and protein expression, and responds differently to stimuli^11^.

There is currently no treatment for post-TBI long-term disorders. Pharmacological strategies targeting modulatory or regulatory processes of NI could prevent the development and progression of long-term disorders and may appear to be promising therapeutic strategies. These last years, microglial activation and its modulation have become an exciting target in the field of NI research. NI monitoring and evaluation of the effects of microglia-targeted strategies need noninvasive technique using specific markers, ideally by *in vivo* imaging. The latter allows a longitudinal monitoring and is transposable from bench to bedside and conversely.

These last decades, the use of positron emission tomography (PET) for imaging microglial activation has appeared as a promising noninvasive method to monitor NI. Among the possible targets for microglia, the 18-kDa translocator protein (TSPO) is the most studied in PET^12^. TSPO is a five transmembrane domain protein located in the outer mitochondrial membrane, discovered in 1977 and previously named “peripheral benzodiazepine receptor”^13^. TSPO has been described as involved in multiple physiological functions such as steroid biosynthesis, membrane biogenesis, heme biosynthesis, cellular proliferation and differentiation, apoptosis^14^. However, recent studies have reported a potential implication of TSPO in inflammatory process and doubts still persist in its precise pharmacological properties^15^. Although it is widely and ubiquitously expressed, its expression in the CNS has been described as mostly restricted to glial cells^16^. Its increased expression during NI, and its colocalization with markers of activated microglia, put it as a reference marker of microglial activation^17–19^. In humans, the increase in TSPO expression has been observed with TEP in stroke, Alzheimer’s and Parkinson’s diseases, amyotrophic lateral and multiple sclerosis, herpetic and HIV encephalopathy^20^. Some studies revealed a long-term increase in TSPO expression, from several months to years after TBI^21,22^.

The historical TSPO radiotracer used for preclinical studies was PK11195, a TSPO ligand^23,24^. But these last years, a new generation of TSPO ligands has been developed: [^11^C]DAA1106, [^18^F]FE-DAA1106, [^11^C]DPA713, [^18^F]DPA714, [^18^F]FEPPA, [^18^F]PBR28, [^18^F]PBR111, [^11^C]SSR18075, [^11^C]CLINME, [^123^I]CLINDE and [^11^C]vinpocetine^25^. These second generation radioligands demonstrate a better brain diffusion, a better affinity for TSPO and a lesser nonspecific binding^26^. [^18^F]FEPPA is one of the newest TSPO PET radiotracers with greater affinity for its target^27^ and one of the two TSPO radioligands being the most advanced in clinical development with [^18^F]DPA714 for PET imaging of neuroinflammation. To date and to our knowledge, only two studies evaluated the use of TSPO radioligand in PET to monitor microglial activation in a TBI model in mice^28,29^.

The purpose of this study was to evaluate the use of the 18-kDa translocator protein (TSPO) in positron emission tomography (PET) and cellular analysis to monitor microglial activation in a mild TBI (mTBI) mouse model.

The use of [^18^F]FEPPA with static PET imaging was evaluated to monitor microglial activation at 1, 3 and 7 days after TBI in mice, as we already evidenced microglial activation in this model^30^. Then, the specificity of TSPO for microglia was evaluated using immunohistochemistry on these brains after TBI. Finally, we investigated and quantified TSPO expression by microglia and other CNS cells, especially infiltrating immune cells and endothelial cells, through immunohistochemistry on brain slices, immunocytochemistry on endothelial and microglial primary cell cultures, and flow cytometry in mouse brain after TBI.

## Results

### [^18^F]FEPPA static PET imaging

**Figure 1:**
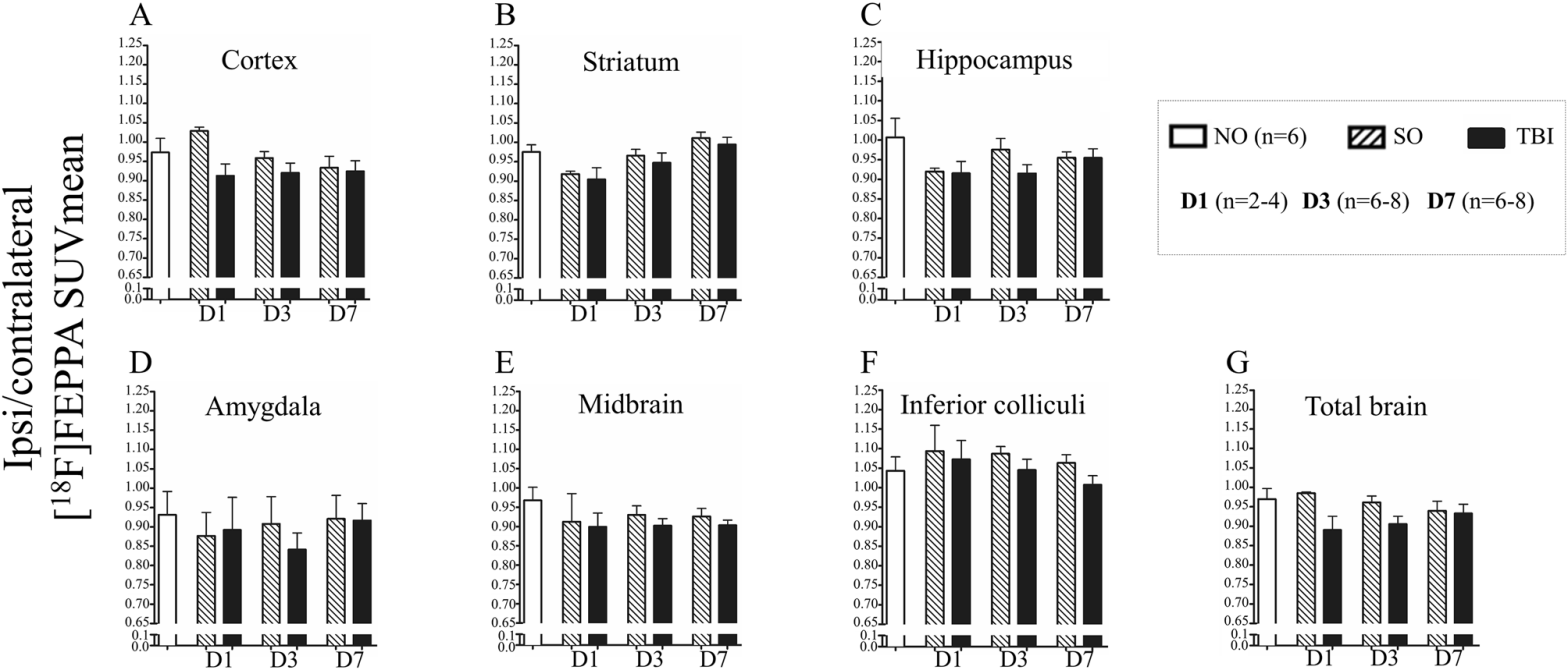
PET ipsi/contralateral [^18^F]FEPPA SUVmean for NO, SO, and TBI mice at D1, D3 and D7. In (**A**) cortex, (**B**) striatum, (**C**) hippocampus, (**D**) amygdala, (**E**), midbrain, (**F**) inferior colliculi and (**G**) the sum of the 6 regions. Data are expressed as means ± S.E.M. Differences were analyzed using a one-way ANOVA. *D* Day, *NO* Non Operated, *SO* Sham-operated, *SUV* standard uptake value, *TBI* traumatic brain injury.

No significant variations of ipsi/contralateral [^18^F]FEPPA SUVmean were observed in the 6 regions studied and their sum, at all studied time-points (Fig. 1 and Supplementary Table S1). Similarly, ipsi/contralateral [^18^F]FEPPA SUVmax did not vary whatever the studied regions and the time-points (data not shown).

### Cellular expression of TSPO in immunohistochemistry and immunocytochemistry

**Figure 2:**
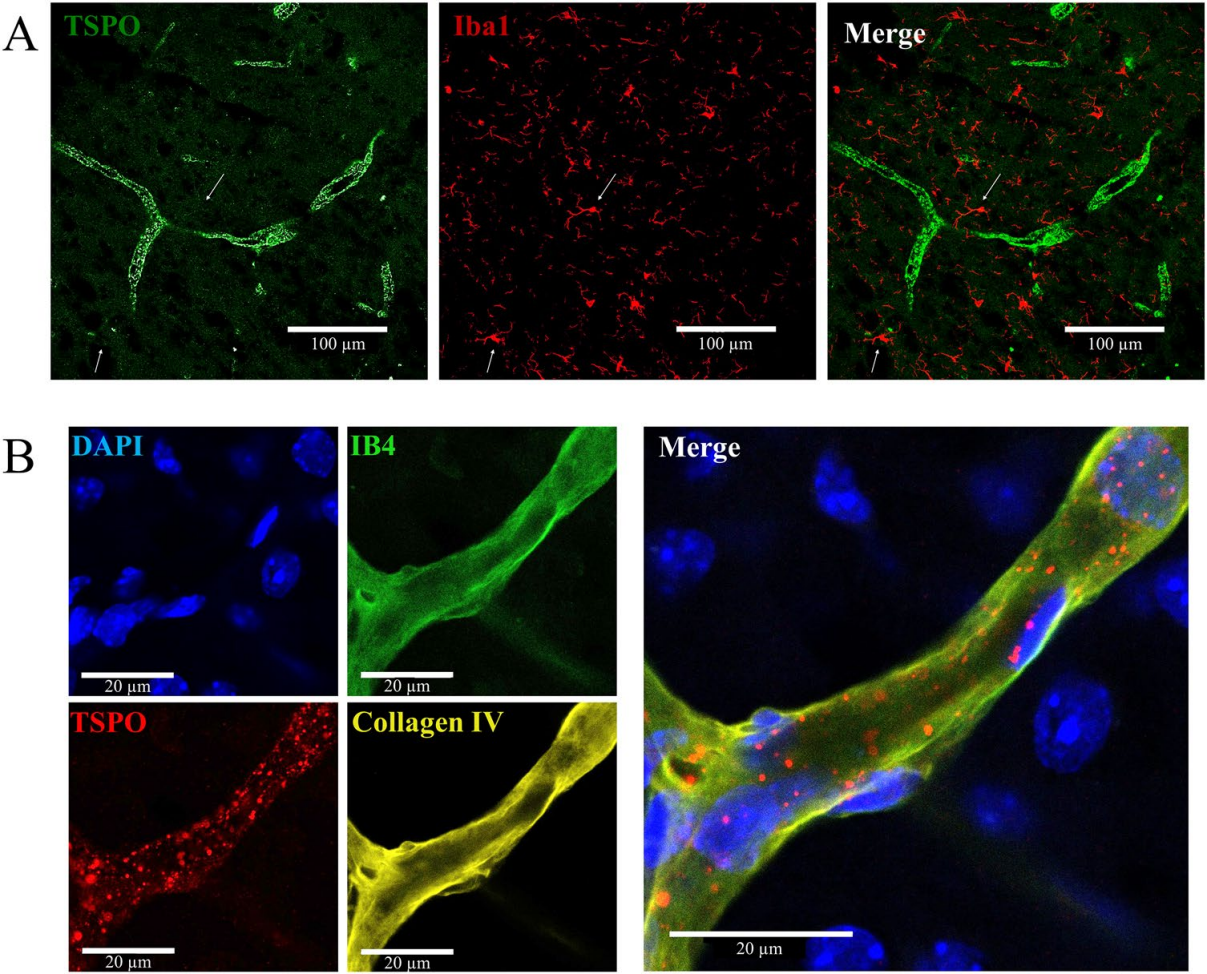
Immunohistochemistry on mouse brain slice 3 days post-TBI around ipsilateral cortical area, (**A**) Iba1/TSPO staining; (**B**) DAPI/TSPO/IB4/Collagen IV staining. *TBI* traumatic brain injury, *TSPO* translocator protein.

In immunohistochemistry, no colocalization was observed between Iba1 and TSPO staining. Moreover, TSPO staining revealed a vascular-like staining different from Iba1 staining (Fig. 2A). This staining was observed in all brain regions, all groups (NO, SO, TBI) and all time-points studied.

To further investigate the vascular-like TSPO staining, immunohistochemistry staining of basal lamina (collagen IV) and endothelial cells (isolectin B4) were performed (Fig. 2B). These staining methods allowed to detail the localization of TSPO as under the basal lamina, revealing an endothelial localization of TSPO (Fig. 2B).

No staining of TSPO with AQP4, specific to astroglia, and the absence of basal-like staining of TSPO revealed that the astrocyte endfeet surrounding the blood vessels did not express TSPO (data not shown).

Finally, the same pattern of staining was observed with an anti-TSPO antibody from a different clone targeting another TSPO epitope.

**Figure 3:**
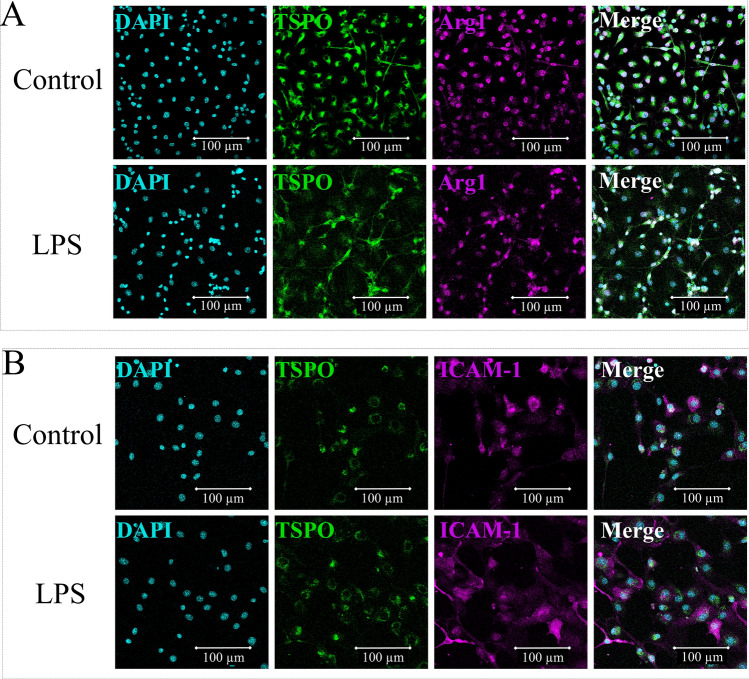
Immunocytochemistry on cell culture. (**A**) primary microglial cell culture with DAPI/Arg1/TSPO staining, treated with PBS (control condition) or LPS 100 ng/mL (LPS), at × 40 lens and (**B**) brain endothelial cell strain (bEnd3) with DAPI/TSPO/ICAM-1 staining, treated with PBS (control condition) or LPS 100 ng/mL (LPS) at × 40 lens. *bEnd3* brain endothelial cell strain, *LPS* Lipopolysaccharide, *PBS* Phosphate Buffered Saline, *TBI* traumatic brain injury, *TSPO* translocator protein.

To evaluate the expression of TSPO by microglia and/or endothelial cells, immunocytochemistry was performed using antibodies raised against TSPO and microglia (Arg1 and Iba1; data not shown for Iba1) or endothelial cell (ICAM-1) specific markers on primary microglial cell cultures and on brain endothelial cell (bEnd3) culture respectively. TSPO was clearly expressed by microglial cells, whether they were stimulated by pro-inflammatory stimulus (LPS) or not (control condition; Fig. 3A). The same colocalization was observed when they were stimulated by anti-inflammatory stimulus (IL-4; data not shown). We also observed the expression of TSPO by bEnd3 cells whether they were stimulated by pro-inflammatory stimulus (LPS) or not (control) (Fig. 3B) showing that endothelial cells also expressed TSPO.

### Immune cell distribution after TBI

Under homeostatic conditions, microglia represented 94.6 ± 1.1% of the total of immune cells, macrophages 0.7 ± 0.1%, monocytes 4.0 ± 1.1%, neutrophils 0.3 ± 0.1% and lymphocytes 0.4 ± 0.1% (Table 1 and Supplementary Fig. S1A). Proportion of microglia was not modified by TBI at one day (93.1 ± 1.9%) and significantly decreased at three days (89.4 ± 1.1%; *P* < 0.05). Conversely, all the other immune cell populations increased at one and three days after a TBI (Table 1), reflecting the peripheral immune cell recruitment following TBI^31^.

**Table 1:** Immune cells distribution in the CNS in non-operated mice and at one and three days after TBI.

	Non-operated (n = 10)	TBI	
		D1 (n = 6)	D3 (n = 8)
Microglia	94.6 ± 1.1%	93.1 ± 1.9%	89.4 ± 1.1% *
Macrophages	0.7 ± 0.1%	2.3 ± 0.6% *	2.8 ± 0.5% **
Monocytes	4.0 ± 1.1%	3.2 ± 1.2%	4.5 ± 1.0%
Neutrophils	0.3 ± 0.1%	0.6 ± 0.1%	2.0 ± 0.5% ***
Lymphocytes	0.4 ± 0.1%	0.8 ± 0.1%	1.4 ± 0,3% **

Data were expressed as mean percentage of the total immune cell population ± S.E.M. Differences were analyzed using a one-way ANOVA followed by a Dunnett’s test. *P < 0.05, **P < 0.01 and ***P < 0.001 *versus* non-operated.

### TSPO expression

**Figure 4:**
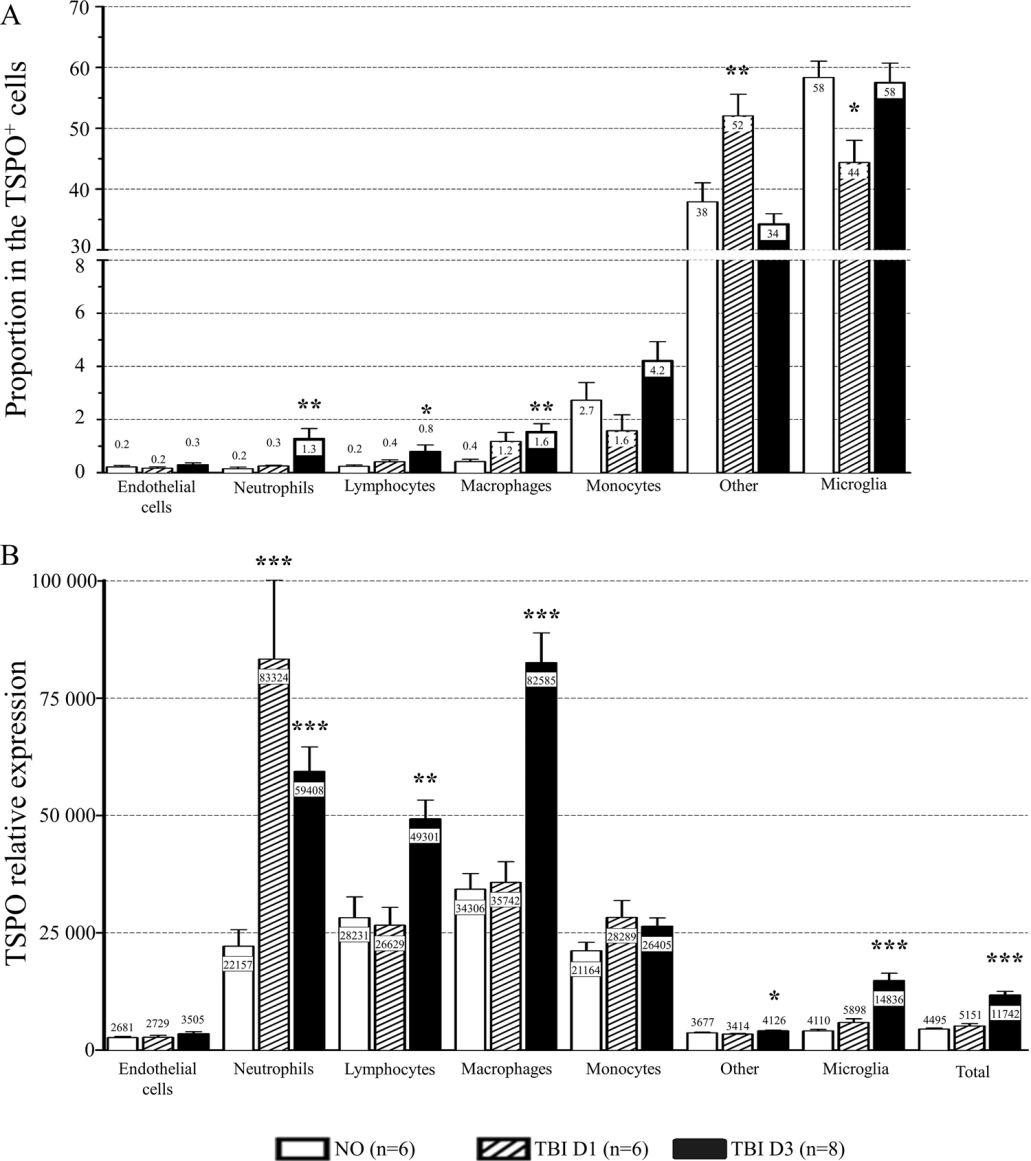
TSPO expression in central nervous system cells. (**A**) Relative percentage of TSPO^+^ cells and (**B**) TSPO expression by cell population in non-operated mice, and at one day and three days after TBI. Data were expressed as mean ± S.E.M. Differences were analyzed using a one-way ANOVA followed by a Dunnett’s test. *P < 0.05, **P < 0.01 and ***P < 0.001 *versus* non-operated. *TBI* traumatic brain injury, *TSPO* translocator protein.

In the homeostatic state, microglia were the main cell type among TSPO^+^ cells (58.3 ± 2.7%) while macrophages (0.4 ± 0.1%), monocytes (2.7 ± 0.7%), lymphocytes (0.2 ± 0.1%), neutrophils (0.2 ± 0.1%) and endothelial cells (0.2 ± 0.1%) represented less than 4% of all TSPO^+^ cells. The rest of TSPO^+^ cells (37.9 ± 3.1%) were not identified (Fig. 4A and Supplementary Table S2 and Fig. S1B) and could be neurons, astrocytes and/or oligodendrocytes. The proportion of microglia among TSPO^+^ cells significantly decreased one day after TBI (44.4 ± 3.6%; *P* < 0.05) and got back to its homeostatic level at 3 days after TBI (57.5 ± 3.2%). At three days, TBI increased the proportion of macrophages (1.6 ± 0.3%, *P* < 0.01), lymphocytes (0.8 ± 0.2%, *P* < 0.05) and neutrophils (1.3 ± 0.4%, *P* < 0.01) within TSPO^+^ cells, demonstrating parenchyma infiltrating blood cells. Finally, the proportion of unidentified cells in TSPO^+^ cells increased at one day (52.0 ± 3.6%; *P* < 0.01) and got back to its homeostatic level at three days (34.3 ± 1.7%) (Fig. 4A and Supplementary Table S2).

The geometric mean of TSPO relative expression intensity was higher in the peripheral immune cells such as macrophages (34 036 ± 3 314, *P* < 0.001 *versus* microglia), monocytes (21 164 ± 1 814, *P* < 0.001 *versus* microglia), lymphocytes (28 231 ± 4 430, *P* < 0.001 *versus* microglia) and neutrophils (22 157 ± 3 508, *P* < 0.001 *versus* microglia) than in microglia (4 110 ± 310). Conversely, the TSPO expression intensity was lower in endothelial cells (2 681 ± 251) and in the rest of unidentified TSPO^+^ cells (3 677 ± 149) than in microglia. Following TBI, TSPO expression of TSPO^+^ cells (4 495 ± 214) did not differ at one day (5 151 ± 511) but was significantly increased at three days after TBI (11 742 ± 786.4; *P* < 0.001). TSPO expression profile of endothelial cells (2 729 ± 431 at D1 and 3 505 ± 426 at D3) and monocytes (28 289 ± 3 594 at D1 and 26 405 ± 1 771 at D3) was not modified by time after TBI. In neutrophils, TSPO expression was significantly higher both at D1 (83 324 ± 16 866, *P* < 0.001) and D3 (59 408 ± 5 214, *P* < 0.05). In all the other cells, the increase was observed only at D3: microglia (5 898 ± 790 at D1 and 14 836 ± 1 560 at D3; *P* < 0.001), macrophages (35 742 ± 4 425 at D1 and 82 585 ± 6 329 at D3; *P* < 0.001), lymphocytes (26 629 ± 3 800 at D1 and 49 301 ± 3 990 at D3, *P* < 0.01) and all the other unidentified cells (3 414 ± 126 at D1 and 4 126 ± 121 at D3; *P* < 0.05) (Fig. 4B and Supplementary Table S3).

In immune cells, 94 to 100% of the cells expressed TSPO (Table 2). Conversely, only 11.5 ± 2.5% of endothelial cells expressed TSPO in non-operated mice. These proportions did not significantly differ after TBI (Table 2).

**Table 2:** Percentage of TSPO^+^ cells in each cellular population in ipsilateral brain of non-operated mice and at one day and three days after TBI.

	Non-operated (n = 10)	TBI	
		D1 (n = 6)	D3 (n = 8)
Microglia	98.7 ± 0.2%	97.8 ± 0.4%	98.4 ± 0.7%
Macrophages	100.0 ± 0.0%	100.0 ± 0.0%	100.0 ± 0.0%
Monocytes	98.1 ± 0.7%	98.1 ± 0.4%	99.6 ± 0.2%
Lymphocytes	94.0 ± 3.2%	98.6 ± 0.6%	93.4 ± 3.9%
Neutrophils	95.3 ± 2.1%	90.1 ± 2.7%	99.6 ± 0.2%
Endothelial cells	11.5 ± 2.5%	9.7 ± 2.7%	17.2 ± 2.2%

Data were expressed as mean of the percentage ± S.E.M. Differences were analyzed using a one-way ANOVA followed by a Dunnett’s test.

## Discussion

Many studies linked NI, characterized by microglial activation, to white matter injuries, after TBI^7,8^. For more than 20 years, TSPO has been described as specific for glial cells in the CNS and is widely used as a reference marker for microglial activation in different pathological context^17–19^. However, the use of TSPO to specifically monitor the microglial activation remains a matter of concern in the literature^12^. In this study, we evaluated the use of TSPO for microglial activation monitoring in an experimental model of TBI in mice, in which we previously highlighted a microglial activation from one to seven days post-TBI^30^. For this purpose, we used different methods: PET imaging, immunohistochemistry, immunocytochemistry and flow cytometry.

Flow cytometry analysis showed an increase in TSPO expression in microglia three days after TBI. As microglial activation has been previously described in our TBI model at this time-point^30^, we can assume that TSPO is overexpressed by activated microglia, as previously described^32,33^. We also highlighted an increase in TSPO expression in the overall population of TSPO^+^ cells in the CNS three days after TBI, consistently with the literature^18,19^. However, the involvement of microglia in the overall overexpression of TSPO in the CNS following TBI remains unclear. Indeed, the expression of TSPO by non-microglial cells and endothelial cells has been known for a long time but only recent studies have reported a non-negligible expression of TSPO by activated astrocytes^34,35^ and endothelial cells^36^. Our results confirmed the importance of non-microglial expression of TSPO, as microglia represent only 58% of the TSPO^+^ cells in the CNS in non-operated mice, and 44 to 58% 1 and 3 days after TBI. Moreover, an important proportion of TSPO^+^ cells (34.3 to 52%) were not identified and were non-immune and non-endothelial cells, namely neurons, astrocytes and/or oligodendrocytes. Flow cytometry analysis could not let us to discriminate neurons, oligodendrocytes and astrocytes, but the immunohistochemistry clearly excluded TSPO expression by astrocytic feet around vessels as we could have suspected it.

This data points the lack of specificity of TSPO for microglia and suggests that microglia contributes to the overall increase in TSPO in the CNS after TBI, but is not the only contributing cell. To our knowledge, we are the first to precisely quantify the proportion of microglia among the TSPO^+^ cells, following TBI. The infiltrating immune cells appear to be strong contributors to TSPO increase following TBI. A recent in vitro flow cytometry study reported an increased expression of TSPO in macrophages in a pro-inflammatory context^35^. Flow cytometry analysis allowed us to confirm it in vivo and to highlight that all the immune cells express TSPO. They accounted for only 3.5 to 7.9% of the TSPO^+^ cells, but they expressed TSPO in a stronger way than microglia, both in homeostatic state (*i.e.* non-operated mice) and in an inflammatory context (*i.e.* TBI mice). Moreover, 94 to 100% of the immune cells expressed TSPO and its expression increased in lymphocytes, neutrophils and macrophages following TBI. Interestingly, except in neutrophils, TSPO expression does not increase at one day post-TBI in peripheral immune cells, whereas the post-traumatic peripheral immune cells infiltration process already started, as macrophages proportion is increased at one day post-TBI. Peripheral immune cells infiltration could be due to increased blood–brain barrier (BBB) permeability after TBI, and to an active recruitment process^37^. Thus, our results could mean that the recruitment process does not activate them in a way they overexpress TSPO, or that the infiltration at one day post-TBI is more due to a BBB alteration than to an active recruitment process.

Immunohistochemistry on brain slices confirmed the lack of specificity of TSPO expression as no colocalization of TSPO and Iba1 was observed whereas immunocytochemistry confirmed the expression of TSPO by microglia both activated and non-activated. Moreover, more precise staining precluded the expression of TSPO by astrocytic feet surrounding vessels and revealed an endothelial localization of TSPO similarly to a recent study^38^. Immunostaining on cell culture allowed us to confirm the expression of TSPO by endothelial cells already reported by others^16,39,40^. However, flow cytometry analysis demonstrated that only 11.5% of the endothelial cells expressed TSPO, and these cells represented 0.2% of TSPO^+^ cells, with a lower expression than other cell types.

PET analysis, using [^18^F]-FEPPA a second-generation TSPO radioligand, did not reveal an increased expression of TSPO 1 and 3 days after TBI, whereas we demonstrated microglial activation and NI in this model^30^. The lack of specificity of TSPO does not explain the absence of increased expression visualized by PET in this TBI model, while an increase was observed in flow cytometry.

Previous study revealed a correlation between [^18^F]-FEPPA signal and the presence of activated microglia^41^. However, this study was performed on a model of Parkinson’s disease on nonhuman primate, which is very tricky to compare with our results. It does mean that the level of neuroinflammation may affect the use of TSPO radiotracer to monitor microglial activation. To our knowledge, to date, only two studies evaluated the use of PET imaging to target TSPO in a TBI mouse model, both using the [^18^F]DPA714 TSPO radioligand in a static analysis^28,29^. However, both used a different TSPO radioligand, the [^18^F]DPA714, which may affect the results. Both highlighted an increase in the radioligand SUV after a TBI, 7 days after the TBI in the cortex^28,29^ and for a longer time in the hippocampus^29^. However, both studies used a more severe TBI model than ours. Hosomi et al*.* used a CCI model to induce TBI, like us, but with higher impact parameters than ours and on C57BL/6 J mice^29^, with smaller brain – and thus more sensitive to the same impact –than Swiss mice. Israel et al*.* used a weight-drop closed head injury model to induce TBI, which generated a variable pattern of focal lesions and diffuse axonal damage associated with a BBB leakage. To analyze their data, the authors classified TBI mice into two groups based on the presence of focal lesions in *postmortem* histology. Their TBI mice group showed visible focal lesion, unlike our TBI mice group. The mice with no visible focal lesion were considered as “mild TBI”. Unlike our TBI mice, these mTBI mice showed neurological impairment at D1, reflecting a more severe TBI than ours, but no significant uptake increase in [^18^F]-DPA714 was observed in this group compared to SO^28^. Then, as a previous study described the lack of sensitivity of some second generation radiotracers to detect mild NI^42^, we can assume that our experimental TBI model may not be severe enough to induce an increase in TSPO detectable in TEP with current TSPO radiotracers and PET resolution. Besides, both studies used different TSPO tracers, which could explain the differences in results.

Moreover, those studies did not clearly link TSPO expression to activated microglia. In immunohistochemistry, TSPO staining was not fully co-localized with Iba1 staining but with CD11b staining^29^, which is not a specific marker for microglia. The ex vivo autoradiography revealed a correlation between [^18^F]-DPA714 uptake and the number of Iba1 + amoeboid cells^28^. It revealed a colocalization of an increased TSPO expression and a microglial activation in a NI context in damaged cerebral regions, but did not precisely quantify the part of TSPO expression assignable to activated microglia. Then, [^18^F]-DPA714 uptake could be due to an alteration of BBB integrity, which had not been assessed, or to a global inflammatory process and the expression from all of the cells implicated in NI.

Finally, macrophage depletion, using peripheral clodronate liposomes, suppressed TSPO uptake in the cortex but increased it in the thalamus^29^. This suggests a stronger implication of peripheral macrophages than activated microglia in TSPO expression in the cortex following TBI. In addition, a recent study reported that the endothelial expression of TSPO is not negligible and it should be necessary to consider it in the kinetic model^36^.

The results of real time-quantitative polymerase chain reaction (RT-qPCR) processed on microglial culture submitted to a pro-inflammatory stimulus by TNFα (the full method and results of RT-qPCR can be found in Supplementary Methods) showed an increase in TSPO expression (4.3 fold), but much lower than other microglial markers (15 and 433 fold, respectively for TNFα and NOS2 mRNA expression). As TSPO seems to be less sensitive than other microglial or NI markers, it strengthens our hypothesis of a too low increase in TSPO expression to be measured in PET (Supplemental Fig. S2).

Besides, TSPO expression was not modified under anti-inflammatory stimulus by IL-4 (data not shown), demonstrating the expression of TSPO by pro-inflammatory M1 microglial phenotype^35,43^.

Finally, further concerns remain on the precise activity of TSPO, as anti-inflammatory and neuroprotective properties have recently been attributed to some TSPO ligands^44^. To date, the pharmacokinetic and pharmacological properties of TSPO radioligands – like their binding site on TSPO and their agonist or antagonist nature^15^ – have been poorly understood. Thus, it questions the use of ligand of TSPO for monitoring microglial activation and NI process.

## Limitations

PET analysis samples may appear small, impeding the detection of a potent slight radiotracer binding variation. However, PET radiotracers need to be sufficiently sensitive to detect a variation with a small number of animals. Moreover, PET imaging is an expensive and challenging to handle method, limiting the number of animals. Immunohistochemical staining on mouse brain sections revealed an exclusive endothelial staining for TSPO. No colocalization was found with microglia or other immune cells, which express TSPO more importantly than endothelial cells, according to our flow cytometry results. The same staining was obtained with two different anti-TSPO antibodies that had different clones. In addition, one of the two antibodies was identical to the one we used in flow cytometry, which effectively labelled the microglia. Finally, our immunocytochemical TSPO staining with this antibody was effective on microglia cultures. This lack of TSPO binding to microglia in immunohistochemistry could be explained by the difficulty to access the mitochondria in this kind of method which allows less effective cell membrane permeabilization than flow cytometry. Then, the quality of the antibody cannot be questioned. Finally, a number of cells expressing TSPO were not identified by our experiments. These cells could be oligodendrocytes, neurons or astrocytes. The non-identification of these cells is a limitation of our study. However, the objective of our flow cytometry experiments was to quantify the proportion of microglia in cells expressing TSPO, in order to evaluate its specificity for microglia in particular. We therefore did not need to identify all the other cell types expressing TSPO, which was not the objective of this work.

## Conclusion

These results question the use of TSPO as a specific marker of microglial activation in a mTBI mouse model. Microglia appear to be the main cells expressing TSPO and therefore the main contributors to its increase, however its expression by a broad array of cell population, some of which with higher expression level, must be taken into account depending on the type of analysis and the objective sought. In cellular analysis following a mTBI, TSPO appears as a better NI marker than an activated microglia marker. In PET analysis, the current TSPO radiotracers and PET resolution might be not sensitive enough to detect microglial activation in a mTBI model.

## Methods

### Animals

Male Swiss mice were supplied by Janvier labs, weighed 28 to 30 g. All care and experiments were in accordance with the ethical approvals stipulated by the Animal Ethics Committee of Paris Descartes University, the French regulations and the European Council Directive of September 22, 2010 (2010/63/EEC) on the protection of animals for experimental use (APAFIS#4765). Animals were housed under temperature (22 ± 2 °C) and light (12 h per day) controlled conditions with access to food and water ad libitum.

The study was carried out in compliance with the ARRIVE guidelines.

### Controlled cortical impact-induced brain injury

The Controlled Cortical Impact (CCI) model was performed as previously described^30^. Mice were anesthetized with isoflurane and placed in a stereotaxic frame. Body temperature was monitored throughout surgery by a rectal probe and maintained at 37.0 ± 0.5 °C with a homeothermic blanket control unit. A 4-mm craniotomy was performed onto the left temporo-parietal cortex centered between the bregma and lambda (Supplemental Fig. S3A), taking care to leave the *dura mater* intact. Injury was delivered using a 3 mm diameter impactor by a pneumatically controlled device (TBI 0310 Impactor, Precision System Instruments) using the following parameters: diameter 3 mm, velocity 3.5 m/s, depth of cortical deformation 1.0 mm and dwell time 50 ms^45^. Following the injury, the skullcap was replaced by applying bone wax and the skin sutured. Sham-operated (SO) mice underwent the same surgery without impact. To recover from anesthesia and prevent post-surgery hypothermia, animals were placed in an incubator set at 30 °C for one hour. Mice were subsequently returned to their home cage. Non-operated (NO) mice had neither surgery nor anesthesia.

### Positron emission tomography

#### Radiochemical synthesis of [18F]FEPPA

Radiosynthesis of [^18^F]FEPPA was performed at the Unité Claude Kellershohn, in Saint-Louis Hospital, Paris (France), as previously described^46^. Briefly, the radiosynthesis of [^18^F]FEPPA was performed using an AllInOne (Trasis) synthesis module and a tosylated precursor for a one-step fluorine nucleophilic aliphatic substitution.

All reagents and solvents were purchased from commercial suppliers (ABX or Sigma-Aldrich) and were used without further purification. Sep-Pak QMA were purchased from ABX. [^18^F]fluoride ion was produced via the [^18^O(p,n)^18^F] nuclear reaction (IBA Cyclone 18/9 cyclotron). Radioactivity of the final product was measured with a dose calibrator (PET DOSE 5 Ci, COMECER).

#### In Vivo PET/CT imaging

PET/CT imaging was performed using Inveon micro PET/CT scanner (Siemens Medical Solutions) designed for small laboratory animals. Mice were anesthetized (isoflurane/oxygen, 2.5% for induction at 0.8–1.5 L/min, and 1–1.5% at 0.4–0.8 L/min thereafter) during injection of [^18^F]FEPPA (9.9 ± 1.5 MBq) in a volume of 0.15 mL (0.22 ± 0.19 nmol of FEPPA) through the tail vein, and during PET/CT acquisitions, as previously described^46^.

The spatial resolution of Inveon PET device was 1.4 mm full-width at half-maximum at the center of the field of view. Images were reconstructed using a 3D ordered subset expectation maximization method including corrections for scanner dead time, scatter radiations, and random.

#### Protocol

In accordance with metabolism studies^46^, the [^18^F]FEPPA concentration decreases rapidly in the plasma, and get stabilized 90 min after the injection. Conversely, brain concentration is barely stable through time. The acquisition was then started 75 min after injection of [^18^F]FEPPA, and lasted for 30 min. Between the injection and the start of the acquisition, anesthesia was stopped.

PET/CT imaging was performed at 1, 3, and 7 days after surgery or TBI. At D1, 8 mice were imaged: 1 NO, 3 SO and 4 TBI (Supplemental Fig. S4). At D3 and D7, 16 mice were imaged: 2 NO, 6 SO and 8 TBI. No animal was excluded from this experiment. All NO mice were pooled for SUV comparison. One SO D1 acquisition failed and could not be used for SUV comparison.

#### Data analysis and modeling

PET/CT images were visually assessed. Then quantitative analysis of PET/CT images was performed by PMOD version 3.806 image analysis software (PMOD Technologies). All values of radioactivity concentrations were normalized by the injected dose and the weight of the mouse, and expressed as percentage of the injected dose per g of tissue (% ID/g; Supplemental Fig. S3B) for the visualization and expressed as standard uptake value (SUV) maximum (SUVmax) and mean (SUVmean) for the quantification.

PET images were automatically rigidly matched with corresponding CT and then cropped to keep only the brain images. CT were automatically rigidly matched with a T2 MRI template (M. Mirrione, included in PMOD software), and then the transformation applied to the PET image was cropped. This method allowed us to use the atlas of the brain corresponding to the T2 MRI template. The atlas allowed us to quantify 6 regions (cortex, striatum, hippocampus, amygdala, midbrain and inferior colliculi) on the left (ipsilateral) and right (contralateral) side, and 7 regions non laterally-differentiated (thalamus, cerebellum, basal forebrain septum, hypothalamus, brain stem, central gray and superior colliculi) (Supplemental Fig. S3B).

The high variability induced by the injection and acquisition protocol prevented us from making a straight comparison of SUV between mice. SUV values were then normalized through a ratio with a SUV value of a reference region for each animal. The ratio between left (ipsilateral) and right (contralateral) side of the 6 side-differentiable regions (cortex, striatum, hippocampus, amygdala, midbrain and inferior colliculi), and the addition of all these regions (total brain) were calculated. The comparison of the 7 non side-differentiable brain regions (thalamus, cerebellum, basal forebrain septum, hypothalamus, brain stem, central gray and superior colliculi) was not possible.

### Immunohistochemistry and immunocytochemistry

The immunohistochemistry and immunocytochemistry protocols are briefly presented in this section. They are fully detailed in supplementary file.

#### Brain preparation

The day after the PET acquisition (Supplemental Error! Reference source not found. S4), mice were transcardially perfused with NaCl followed by a fixative solution. Brains were removed and kept frozen at -80 °C. Coronal brain Sects. (20 μm thick) were taken using a cryostat (JUNG CM3000, Leica Microsystems). Sections were fixed in acetone for 5 min and then rehydrated in phosphate‐buffered saline (PBS) for 15 min, before processing to immunostaining.

#### Cell culture

Transformed mouse brain endothelial cells bEnd.3 (ATCC CRL-2299) were purchased from Sigma-Aldrich and fixed for 12 min using 4% paraformaldehyde in PBS prior to immunostaining.

Primary mixed glial cell culture was prepared from cortices of postnatal (day P0 to P3) mice as previously described^47,48^ and fixed for 12 min using 4% paraformaldehyde in PBS.

#### Immunostaining

Slices were incubated overnight at 4 °C with the primary antibodies and 90 min with secondary antibodies. Then, slices were incubated with DAPI (Calbiochem; 268,298; 1:20 000) for 90 min at room temperature. For the DAPI/IB4/collagen IV/TSPO staining, a 90-min incubation with a FITC-marked Isolectin B4 from *Bandeiraea simplicifolia* (*Griffonia simplicifolia*) (Sigma, L2895, 1:100) was performed. Antibodies references and dilutions are listed in the Supplemental Table S4 and Supplemental Table S5.

The slices were examined under a SP8 laser scanning confocal microscope (Leica Microsystems).

### Flow cytometry

#### Animals

The objective of the flow cytometry experiment was to evaluate the expression of TSPO by microglial cells, in the homeostatic state and the inflammatory context of our TBI model, whether the inflammation originated from the surgery or the TBI. Thus, TSPO expression in SO mice would not have been useful data. Considering also ethical issues, we included and analyzed only two categories of animals (NO and TBI; 10 NO, 8 TBI at 1 day and 8 at 3 days; Supplemental Fig. S4).

#### Brain dissociation

Brain dissociation and immunostaining protocols are briefly presented in this section. They are fully detailed in supplementary file.

At 1 and 3 days after TBI, or surgery, mice were anesthetized and transcardially perfused with 0.9% NaCl saline. The brains were removed and each hemisphere represented a sample.

Each sample was then dissociated in a mix from the Miltenyi Biotec’s Adult Brain Dissociation Kit (130–107-677), following the manufacturer’s instructions. The cells obtained from the samples were counted and the volume adjusted to obtain 50 to 100.10^6^ cells/mL.

#### Data acquisition

Cells were analyzed with LSRFortessa cell analyzer (BD Biosciences) and data analyzed with FACSDiva (Becton Dickinson) and FlowJo (version 10.5.3; Tree Star) softwares.

After isolating single living cells (Supplemental Fig. S5A):

- monocytes and neutrophils were gated on their Ly-6G and Ly-6C expression profile,
- lymphocytes, macrophages and microglia were gated on their CD45 and CD11b expression profile,
- and endothelial cells were gated on their CD144 expression profile (Supplemental Fig. S5B).

After gating the TSPO^+^ cells on single live cells, the same gating strategy was applied (Supplemental Fig. S5C). This allowed us to quantify the proportion of the different immune cells and endothelial cells in cells expressing TSPO in the CNS, and its relative expression level.

The number of each immune cell population (monocytes, neutrophils, macrophages, lymphocytes and microglia) was counted and added to determine the total number of immune cells. The proportion of each immune cell was calculated by the ratio of the number of the specific immune cell population over the total number of immune cells.

In each isolated cell population, the proportion of TSPO^+^ cells was calculated, and in each cellular population, the TSPO expression was quantified using geometric mean intensity.

### Statistical analysis

Data were expressed as mean ± SEM of *n* observations, where *n* represents the number of animals. All figures and statistical analyses were created with GraphPad Prism 5.0 (Graphpad Software). Values of probability lower than 5% (*P* < 0.05) were considered significant.

To proceed to TEP data analysis, SUVmean and SUVmax were extracted from PMOD software (PMOD Technologies) and converted into Microsoft Excel format.

SUVmean and SUVmax of each of the 6 side-differentiable brain regions were compared using one-way ANOVA followed by a Dunnett’s test.

Immunostaining quantification values were using a Welsh t test as the variance were significantly different.

The proportion of immune cells measured in flow cytometry, and the geometric mean of the TSPO staining intensity were compared using a one-way ANOVA followed by a Dunnett’s test.

## Supplementary Information

Supplementary Information.

## Data Availability

The datasets used and analyzed during the current study are available from the corresponding author on reasonable request.
