# Supplementary material for: From positron emission tomography to cell analysis of the 18-kDa Translocator Protein in mild traumatic brain injury
Source: Sci Rep. 2021 Dec 14;11:24009. doi: 10.1038/s41598-021-03416-3 (PMC8671393; doi:10.1038/s41598-021-03416-3)
Supplement: Supplementary file 1 — Supplementary Information. [file 41598_2021_3416_MOESM1_ESM.docx]

**Supplementary information of: “From positron emission tomography to cell analysis of the 18-kDa Translocator Protein in mild traumatic brain injury”**.

**Clément DELAGE^1,2^, Nicolas VIGNAL^2,3,4^, Coralie GUERIN^5,6^, Toufik TAIB^1^, Clément BARBOTEAU^2^, Célia MAMMA^1^, Kahina KHACEF^1^, Isabelle MARGAILL^1,7^, Laure SARDA-MANTEL^3,4^, Nathalie RIZZO-PADOIN^4,8^, Fortune HONTONNOU^4,9^, Catherine MARCHAND-LEROUX^1,2^, Dominique LEROUET^1,2^, Benoit HOSTEN^2,4,10^, Valérie BESSON^1,2^**

*^1^ Université Paris Descartes, EA4475 - Pharmacologie de la circulation cérébrale, Faculté de Pharmacie de Paris, Paris, France*

*^2^ Université de Paris, Inserm UMR-S 1144, Faculté de Pharmacie de Paris, Paris, France*

*^3^ Assistance Publique – Hôpitaux de Paris (AP-HP), Service de Médecine Nucléaire, Hôpital Lariboisière, Paris, France*

*^4^ Université de Paris, Institut de Recherche Saint-Louis, Unité Claude Kellershohn, Paris, France*

*^5^ Université de Paris, Innovative Therapies in Haemostasis, Inserm, F-75006 Paris, France, Institut Curie, Cytometry Core F-75005, Paris, France*

*^6^ Université de Paris, Inserm UMS 3612 CNRS – US25 Inserm –Faculté de Pharmacie de Paris, Paris, France*

*^7^ Université de Paris, Inserm UMR-S 1140, Faculté de Pharmacie de Paris, Paris, France*

*^8^ CHU de Martinique, Service Pharmacie, Hôpital Pierre Zobda-Quitman, Fort-de-France, France*

*^9^ Université de Paris, Inserm UMR-S 942, hôpital Lariboisière, Paris, France*

*^10^ Assistance Publique – Hôpitaux de Paris (AP-HP), Service Pharmacie, Hôpital Saint-Louis, Paris, France*

**SUPPLEMENTARY METHODS**

**Real time-quantitative polymerase chain reaction (RT-qPCR) for TSPO expression in primary microglial cell culture**

Total RNA was isolated, from primary microglia culture, with the RNeasy Micro Kit according to the manufacturer’s instruction (Qiagen). RNA quality and concentration were assessed by spectrophotometry with the NanodropTM1000 (Thermofischer Scientific). Total RNA was then subjected to reverse transcription using the iScriptTM cDNA synthesis kit (Bio-Rad). Using SYBER Green Super-mix (Biorad), RT-qPCR was performed in triplicate for each sample for 40 cycles with a two-step program (5 seconds of denaturation at 95°C and 10 seconds of annealing at 60°C). Amplification specificity was evaluated with a melting curve analysis. Primers were designed using Primer3 software. Sequence and their NCBI references are given in **Supplemental** **Table S6**. The relative expression of gene of interest was normalized to that of ribosomal protein L13A (Rpl13a), used as a reference gene. For primary microglia culture, the data are presented as relative RNA units with respect to the control group (normalized to 100%).

**Immunohistochemistry and immunocytochemistry**

*Brain preparation*

Mice were anesthetized with sodium pentobarbital, i.p., and transcardially perfused with 0.9% NaCl saline for 2 minutes, followed by a fixative solution (4% paraformaldehyde – PFA) for 4 minutes with a pressure of 100 mmHg. We preferred perfusion at constant pressure and time, rather than constant volume, to avoid cell damage caused by too high pressure and to limit the fixation time which could alter the quality of the immunostaining. The volumes were therefore not fixed and defined but were approximately 20 mL for NaCl and 30 mL for fixative solution.

Brains were removed and post-fixed for one hour, then stood in 50 mL of 20% sucrose for 48h at +4°C. Then, brains were snap frozen for 5 minutes (in 50 mL of 2-methylbutan, -40°C) and kept frozen at -80°C. Coronal brain sections (20 μm thick), were taken from the rostral to caudal part of the brain, using a cryostat (JUNG CM3000, Leica Microsystems), set on gelatinized slides and stored at −80°C. Sections were fixed in acetone on dry ice for 5 minutes, then rehydrated in phosphate‐buffered saline (PBS – NaCl 137 mM, KCl 2.7 mM, Na_2_HPO_4_ 8.1 mM, KH_2_PO_4_ 1.5 mM) for 15 minutes, before processing to immunohistochemistry.

*Cell culture*

Transformed mouse brain endothelial cells bEnd.3 (ATCC CRL-2299, Manassas, Virginia, USA) purchased from Sigma (Sigma-Aldrich, Saint Quentin Fallavier, France) were cultivated in Dulbecco's modified Eagle's medium (DMEM) supplemented with 2 mM glutamine, 10% fetal calf serum, 1 mM sodium pyruvate, 1% non-essential amino acids, 50 U/ml penicillin and 50μg/ml streptomycin in a humidified 5% CO2 incubator at 37 °C. Cells were cultured on glass coverslips coated with poly-D-lysine in 24well plates at a density of 200,000 cells/mL. Some cell wells were treated with lipopolysaccharide (LPS; 5 µg/mL) or tumor necrosis factor-α (TNF-α; 20 ng/mL) or H_2_O_2_ (0.5 and 1 nmol/L). The remaining cells were used as controls. After 24 hours, cells were washed in 500 µL PBS and then fixed with 400 µL 4% PFA for 12 minutes at room temperature, followed by 3×5 minutes of rinsing with 500 µL PBS.

Primary mixed glial cell culture was prepared from cortices of postnatal (day P0 to P3) mice. Brains were harvested and dissected into ice-cold DMEM-High Glucose (HG, 4,5g/L) medium supplemented with 2% of antibiotic solution (penicillin 10 000 U/ml - streptomycin 10 mg/ml), and the meninges were gently removed. Cortices were collected, and then mechanically dissociated and suspended in DMEM-HG medium supplemented with 10% of heat-inactivated fetal bovine serum, 2 mM of L-glutamine and 0.01% of antibiotic solution. Glial cells were cultured in poly-D-lysine coated flasks under humidified atmosphere containing 5% CO_2_ at 37°C. Fourteen days later, microglia were isolated from the mixed glial population by an orbital shaking (140 rpm, 37°C) of the flasks for 30 minutes. The supernatants containing microglia were collected and centrifuged (1 200 *g* x 10 minutes, 4°C). The pellets were re-suspended in fresh DMEM-HG medium supplemented with 10% of heat-inactivated fetal bovine serum, 2 mM of L-glutamine and 0.01% of antibiotic solution. Microglia were seeded onto 12- or 24-well culture plates, previously treated with poly-D-lysine, at a density of 400 000 cells/ml for protein or ribonucleic acid (RNA) collection, respectively. For immunofluorescence staining, cells were seeded on poly-D-lysine coated coverslips placed in 24-well plates. One day after plating, some cell wells were treated with LPS (100 ng/mL) or IL-4 (20 ng/mL). The remaining cells were used as controls. After 24 hours, cells were washed in 500 µL PBS and then fixed with 400 µL 4% PFA for 12 minutes at room temperature, followed by 3×5 minutes of rinsing with 500 µL PBS.

The culture purity, determined by immunofluorescence labeling using ionized calcium binding adapter molecule 1 (Iba-1, a specific marker of microglia) antibody (Abcam, ab5076), is about 98%.

*Immunostaining*

The slides were surrounded by Dakopen to maintain the antibody solutions on the slide during incubation.

Slices were incubated overnight at 4°C in 150 µL of primary antibodies, diluted in PBS – gelatin (0.2%) – Triton X-100 (0.25%). Following incubation in primary antibody, slices were washed 3 × 5 minutes with 100 mL of PBS-Tween 0.1%, then incubated in 150 µL of secondary antibodies diluted in PBS – gelatin (0.2%) – Triton X-100 (0.25%). After 90 min of incubation, slices were washed 3 x 5 minutes with 100 mL of PBS, then incubated with 150 µL of DAPI (Calbiochem; 268298; 1:20 000) for 90 minutes at room temperature. Finally, slices were washed 3 x 5 minutes with 100 mL of PBS. For the DAPI/IB4/collagen IV/TSPO staining, a 90-minutes incubation with 150 µL of FITC-marked Isolectin B4 from *Bandeiraea simplicifolia* (*Griffonia simplicifolia*) (Sigma, L2895, 1:100) was performed. Antibodies references and dilutions are listed in the **Supplemental Table S4** and **Supplemental Table S5**.

For each experimentation, a negative control was performed on a slide identical to the one already used in the manipulation. The "negative control" slides followed the same protocol as the other slides except for the incubation with primary antibodies, during which they were incubated with 150µL of the PBS-gelatin (0.2%)-triton X-100 (0.25%) solution without antibodies.

Then, the slides were washed 3 x 5 minutes with 100 mL of PBS and mounted with Fluoromount-G (SouthernBiotech) and examined under a SP8 laser scanning confocal microscope (Leica Microsystems).

**Flow cytometry**

*Brain preparation*

Mice were anesthetized with 150 mg/kg sodium pentobarbital, i.p.. They were transcardially perfused with 0.9% NaCl saline for 4 minutes at a pressure of 100 mmHg. We preferred processing perfusion at constant pressure and time, rather than constant volume, to avoid cell damage caused by too high pressure. The volumes were therefore not fixed and defined but were approximately 40 mL for NaCl. The brains were then quickly removed and the hemispheres separated from each other. Each hemisphere represented a sample and was cut into small pieces and placed in 2 mL ice-cold HBSS (Hanks' balanced salt solution; Gibco; 14185-052) until all brains were removed.

Samples were transferred to tubes containing 1.980 µL of the enzymatic digestion mixture prepared using solutions from a commercial kit (Adult Brain Dissociation Kit, Miltenyi Biotec, Germany) according to the manufacturer's instructions. Tissue dissociation was performed at 37°C in a water bath for 40 minutes with manual mechanical trituration. Samples were centrifuged (1000 g, 10 minutes, 4°C), the pellet resuspended in PBS (Gibco; 70011-036) and the suspension passed through a 70 µm filter (MACS SmartStrainers; Miltenyi Biotec; 130-110-916) to remove undissociated tissue pieces. Samples were centrifuged (1,000 g, 10 minutes, 4°C) and the pellet resuspended in a solution of PBS (at 4°C) and tissue debris removal solution contained in the Adult Brain Dissociation Kit (Miltenyi Biotec; Germany). Four milliliters of PBS at 4°C were gently added to the surface and the sample centrifuged (3000 g, 10 minutes, 40°C), resulting in the formation of 3 phases. Only the lower phase, containing the cell suspension, was retained, resuspended in PBS at 4°C and centrifuged (1,000 g, 10 min, 4°C). After resuspending the pellet in 1 mL of PBS at 4°C, the cells were counted using a Malassez cell and PBS was added to adjust the concentration to 50 to 100 million cells per mL (i.e., 10 to 20 million glial cells per mL).

*Immunostaining*

For each sample, 100µL were proceeded to immunostaining. Firstly, samples were incubated with 10 μL of Zombie NIR (Biolegend, 423105, 1: 500 in PBS), then rinsed with 500 μL of FACS buffer (2% bovine serum albumin, 5mM ethylenediaminetetraacetic acid in PBS; BSA, Miltenyi Biotec; 130- 091- 376; EDTA, Sigma, ED4SS), and centrifuged at 800 g for 6 minutes. Control samples without Zombie NIR labeling were incubated with 10 μL of PBS.

For multicolor staining, samples were incubated with 90 μL of TruStain FcX (Biolegend, 101320, 1:10 in FACS buffer) 2 minutes to reduce non-specific bindings and then with 10μl of fluorochrome-conjugated antibodies solution or their corresponding isotopic controls diluted in FACS buffer for 30 minutes. Samples were then washed with 1 mL of FACS buffer and centrifuged (800g, 5 minutes) twice. Antibodies concentrations and references are listed in **Supplemental Table S7**.

**SUPPLEMENTARY RESULTS**

**Real time-quantitative polymerase chain reaction (RT-qPCR) for TSPO expression in primary microglial cell culture**

TSPO expression significantly increased in microglia under pro-inflammatory stimulus (4.3 fold; p<0.01) but to a lesser extent compared to NOS2 (433 fold; p<0.0001) and TNFα (15 fold; p<0.001) (**Supplemental** **Figure S2**).

**SUPPLEMENTARY TABLES**

|  | NO  (n=6) | D1 | | D3 | | D7 | |
| --- | --- | --- | --- | --- | --- | --- | --- |
|  |  | **SO**  **(n=2)** | **TBI**  **(n=4)** | **SO**  **(n=6)** | **TBI**  **(n=8)** | **SO**  **(n=6)** | **TBI**  **(n=8)** |
| Cortex | 0.97 ± 0.04 | 1.03 ± 0.01 | 0.91 ± 0.03 | 0.96 ± 0.02 | 0.92 ± 0.03 | 0.93 ± 0.03 | 0.92 ± 0.03 |
| Striatum | 0.98 ± 0.02 | 0.92 ± 0.01 | 0.90 ± 0.03 | 0.97 ± 0.02 | 0.95 ± 0.03 | 1.01 ± 0.02 | 0.99 ± 0.02 |
| Hippocampus | 1.01 ± 0.05 | 0.92 ± 0.01 | 0.92 ± 0.03 | 0.98 ± 0.03 | 0.92 ± 0.02 | 0.96 ± 0.02 | 0.96 ± 0.02 |
| Amygdala | 0.93 ± 0.06 | 0.88 ± 0.06 | 0.89 ± 0.08 | 0.91 ± 0.07 | 0.84 ± 0.04 | 0.92 ± 0.06 | 0.92 ± 0.04 |
| Midbrain | 0.97 ± 0.04 | 0.91 ± 0.07 | 0.90 ± 0.04 | 0.93 ± 0.02 | 0.90 ± 0.02 | 0.93 ± 0.02 | 0.90 ± 0.01 |
| Inferior colliculi | 1.04 ± 0.04 | 1.09 ± 0.07 | 1.07 ± 0.05 | 1.09 ± 0.02 | 1.05 ± 0.03 | 1.06 ± 0.02 | 1.01 ± 0.02 |
| Total brain | 0.97 ± 0.03 | 0.99 ± 0.00 | 0.89 ± 0.04 | 0.96 ± 0.02 | 0.91 ± 0.02 | 0.94 ± 0.02 | 0.93 ± 0.02 |

**Table S1.** PET ipsi/contralateral [^18^F]FEPPA SUVmean for NO, SO, and TBI mice at 1, 3 and 7 days. Data were expressed as means ± S.E.M. Differences were analyzed using a one-way ANOVA.

|  | Non-operated  (n=10) | TBI | |
| --- | --- | --- | --- |
|  |  | **D1 (n=6)** | **D3 (n=8)** |
| Microglia | 58.3 ± 2.7% | 44.4 ± 3.6% * | 57.5 ± 3.2% |
| Macrophages | 0.4 ± 0.1% | 1.2 ± 0.3% | 1.6 ± 0.3% ** |
| Monocytes | 2.7 ± 0.7% | 1.6 ± 0.6% | 4.2 ± 0.7% |
| Lymphocytes | 0.2 ± 0.1% | 0.4 ± 0.1% | 0.8 ± 0.2% * |
| Neutrophils | 0.2 ± 0.1% | 0.3 ± 0.1% | 1.3 ± 0.4% ** |
| Endothelial cells | 0.2 ± 0.1% | 0.2 ± 0.1% | 0.3 ± 0.1% |
| Others | 37.9 ± 3.1% | 52.0 ± 3.6% ** | 34.3 ± 1.7% |

**Table S2.** TSPO^+^ cell distribution in ipsilateral brain of non-operated mice and at one day and three days after TBI. Data were expressed as means of the percentage of cell population on total TSPO^+^ cells ± S.E.M. Differences were analyzed using a one-way ANOVA followed by a Dunnett’s test. *p<0.05, **p<0.01 and ***p<0.001 *versus* non-operated.

|  | Non-operated  (n=10) | TBI | |
| --- | --- | --- | --- |
|  |  | **D1 (n=6)** | **D3 (n=8)** |
| Total | 4 495 ± 214 | 5 151 ± 511 | 11 742 ± 786 *** |
| Microglia | 4 110 ± 310 | 5 898 ± 790 | 14 836 ± 1 560 *** |
| Macrophages | 34 036 ± 3 314 ††† | 35 742 ± 4 425 | 82 585 ± 6 329 *** |
| Monocytes | 21 164 ± 1 814 ††† | 28 289 ± 3 594 | 26 405 ± 1 771 |
| Lymphocytes | 28 231 ± 4 430 ††† | 26 629 ± 3 800 | 49 301 ± 3 990 ** |
| Neutrophils | 22 157 ± 3 508 ††† | 83 324 ± 16 866 *** | 59 408 ± 5 214 *** |
| Endothelial cells | 2 681 ± 251 | 2 729 ± 431 | 3 505 ± 426 |
| Others | 3 677 ± 149 | 3 414 ± 126 | 4 126 ± 121 * |

**Table S3.** TSPO relative expression in each isolated TSPO^+^ cellular population in ipsilateral brain of non-operated mice and at one day and three days after TBI. Data were expressed as mean of the relative expression per cells ± S.E.M. Differences were analyzed using a one-way ANOVA followed by a Dunnett’s test. *p<0.05, **p<0.01 and ***p<0.001 *versus* non-operated. †††<0.001 *versus* microglia of non-operated.

| Immunogen | Target species | Host Species | Supplier | Reference | Dilution |
| --- | --- | --- | --- | --- | --- |
| Iba1 | Mouse | Goat | Abcam | ab5076 | 1:500 |
| TSPO | Mouse | Rabbit | Abcam | ab109497 | 1:500 |
| TSPO | Mouse | Rabbit | Invitrogen | SA90-03 | 1:150 |
| Collagen IV | Mouse | Goat | SouthernBiotech | 1340-01 | 1:200 |
| ICAM-1 | Mouse | Goat | R&D Systems | AF796 | 1:20 |
| Arg1 | Mouse | Goat | Santa Cruz Biotechnologies | Sc-18355 | 1:200 |

**Table S4**. List of primary antibodies for immunohistochemistry and immunocytochemistry.

| Fluorochrome | Target species | Host Species | Supplier | Reference | Dilution |
| --- | --- | --- | --- | --- | --- |
| AF 488 | Rabbit | Donkey | Molecular Probes | A21206 | 1:1 000 |
| Cy3 | Goat | Donkey | Jackson Immuno Research | 705-165-147 | 1:1 000 |

**Table S5**. List of secondary antibodies for immunohistochemistry and immunocytochemistry.

| **Target gene** | **Forward** | **Reverse** |
| --- | --- | --- |
| NO synthase 2 (*Nos2*) | CCC TTC AAT GGT TGG TAC ATG G | ACA TTG ATC TCC GTG ACA GCC |
| Translocator Protein (*mTspo*) | AGA GGA CGC TAT GGT TCC CTT | GCC ACC CCA CTG ACA AGC |
| Tumor necrosis factor alpha (*Tnfα*) | GCC TCT TCT CAT TCC TGC TT | AGG GTC TGG GCC ATA GAA CT |
| Ribosomal protein L13 (*Rpl13*) | ACA GCC ACT CTG GAG GAG AA | GAG TCC GTT GGT CTT GAG GA |

**Table S6.** Primer sequences

| Target/immunogen | Fluorophore | Supplier | Reference | Dilution |
| --- | --- | --- | --- | --- |
| CD11b | PE/Dazzle 594 | Biolegend | 101255 | 1:3000 |
| CD45 | BV 785 | Biolegend | 103149 | 1:160 |
| CD144 | BV 421 | Biolegend | 129605 | 1:160 |
| Ly-6C | BV 605 | Biolegend | 128036 | 1:200 |
| Ly-6G | PE/Cy7 | Biolegend | 127617 | 1:120 |
| TSPO | AF647 | Abcam | ab199836 | 1:1000 |

**Table S7**. List of antibodies for flow cytometry.

**SUPPLEMENTARY FIGURES**

**
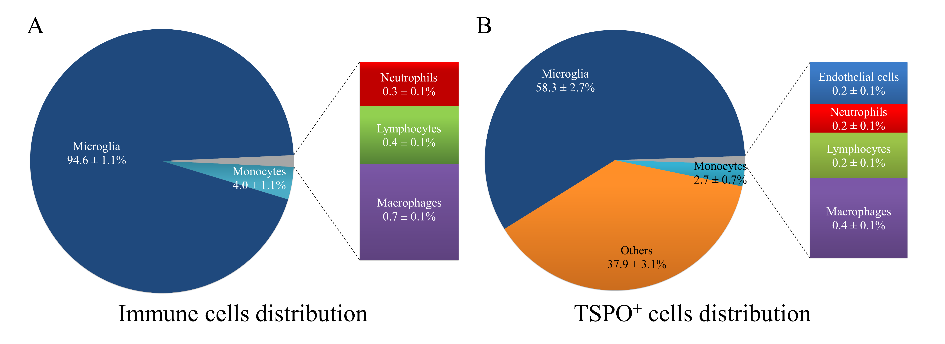
**

**Figure S1**. Distribution of (**A**) immune cells and (**B**) TSPO^+^ cells in the central nervous system.

Distribution at the homeostatic state. Data were expressed as mean ± S.E.M. (n=10).

*Abbreviations: TSPO: Translocator Protein.*


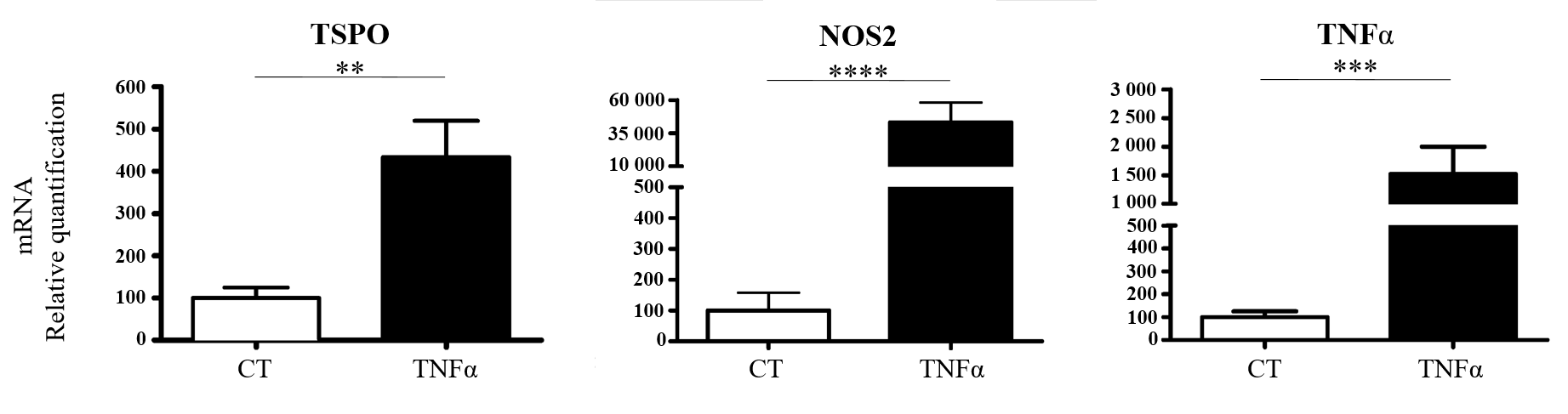
 **Figure S2.** Microglial gene expression under pro-inflammatory stimulus (TNFα 50 ng/mL), mRNA shown relative to control (CT; normalized to 100%). Data were expressed as means ± S.E.M. Differences were analyzed with paired t test with a Welsh’s correction. *p<0.05; **p<0.01; ***p<0.001 and ****p<0.0001.

**
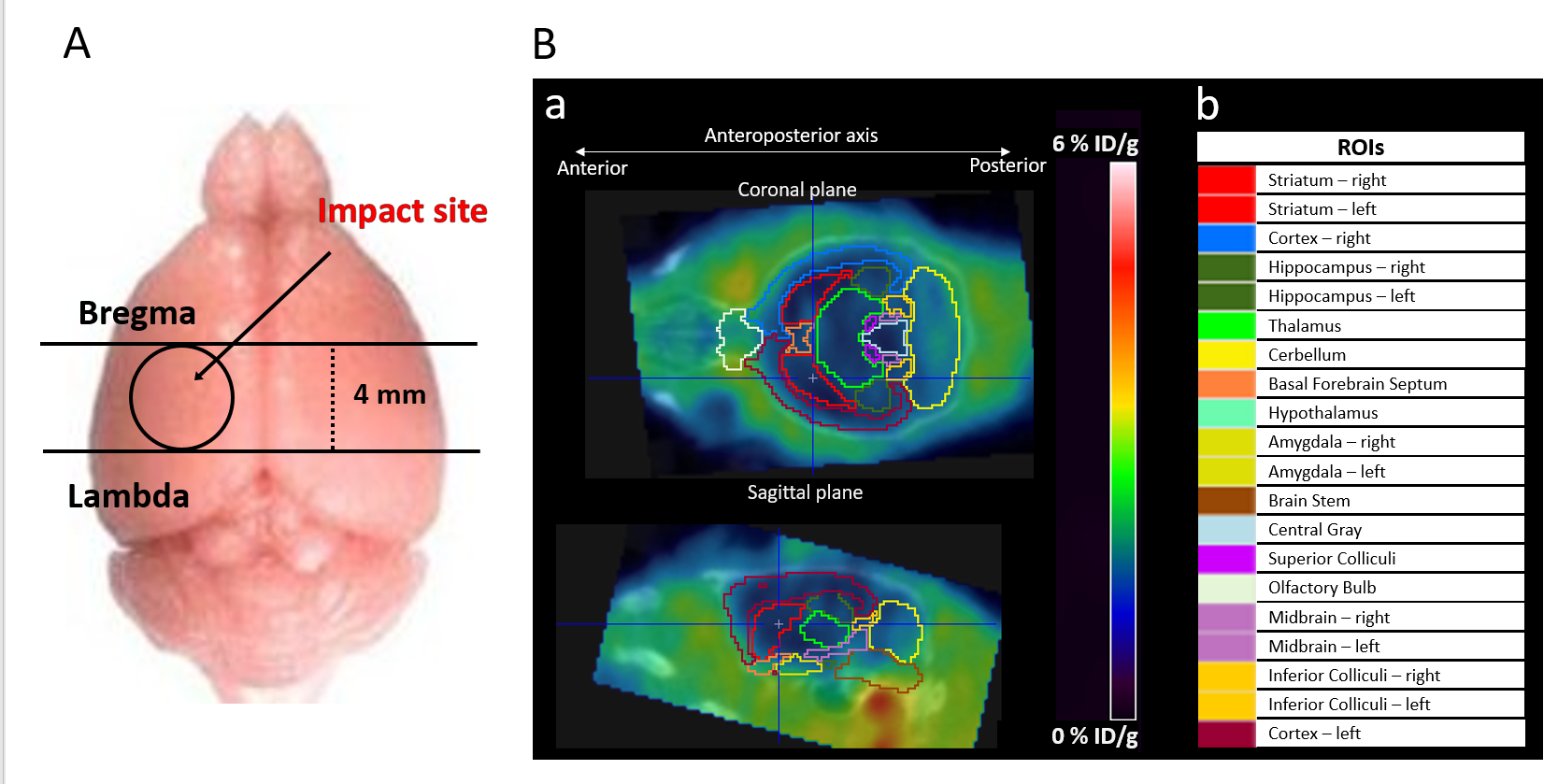
**

**Figure S3.** **A.** Area of the cortical impact; **B.** PET/CT image of [^18^F]FEPPA binding (from PMOD Software v3.806) (**a**) expressed in %ID/g and viewed in a coronal and sagittal plane, with (**b**) the atlas of the ROI used for quantification.

**
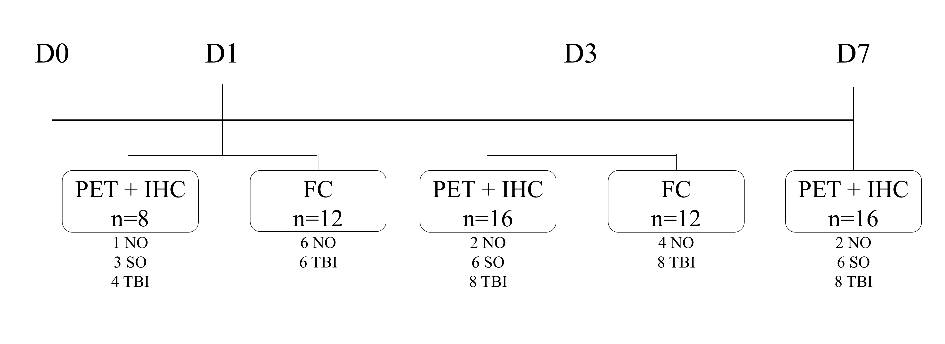
**

**Figure S4.** PET, IHC and FC protocols.

For PET acquisitions, 8 mice (1 NO, 3 SO and 4 TBI) at D1 and 16 mice (2 NO, 6 SO and 8 TBI) at D3 and D7 were imaged. These mice were then used for IHC. For FC analysis, 12 mice (6 NO and 6 TBI) were used at D1 and 12 mice (4 NO and 8 TBI) at D3.

*Abbreviations: D: Day; FC: Flow Cytometry; IHC: Immunohistochemistry; NO: Non Operated; PET: Positron Emission Tomography; SO: Sham Operated; TBI: Traumatic Brain Injury.*

**
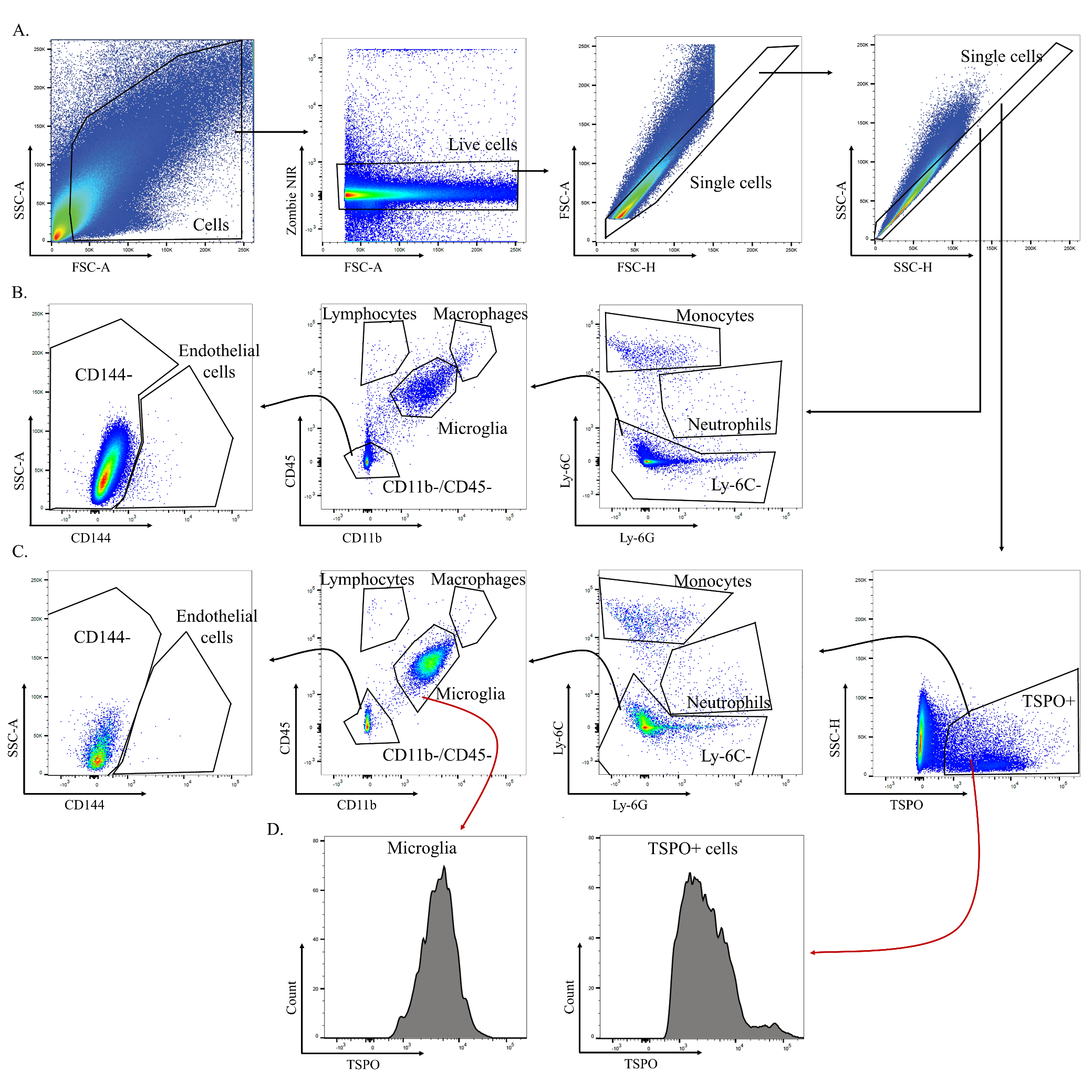
**

**Figure S5.** Flow cytometry gating strategy to sort the different cellular populations.

Cells were distinguished (**A**) from debris using forward (FSC-A) and side (SSC-A) scatters, followed by cell doublet and aggregate elimination (FSC-A/FSC-H and SSC-A/SSC-H). Dead cells were gated out by strong positivity for the dead cell discrimination marker Zombie Dye. (**B**) Monocytes (Ly-6G^-^/Ly-6C^hi^) and neutrophils (Ly-6G^+^/Ly-6C^int^) were gated. In the other cells (Ly-6C^-^), lymphocytes (CD11b^-^/CD45^hi^), macrophages (CD11b^+^/CD45^hi^) and microglia (CD11b^+^/CD45^int^) were gated. Finally, endothelial cells were gated with CD144^+^, in the other cells (CD11b^-^/CD45^-^). (**C**) The same gating strategy was applied to TSPO^+^ cells. (**D**) TSPO expression was quantified in each cellular population using geometric mean intensity.
